# Clinical and Physiological Variables in Patients with Post-COVID-19 Condition and Persistent Fatigue

**DOI:** 10.3390/jcm13133876

**Published:** 2024-06-30

**Authors:** Maércio Santos, Mariana Dorna, Estefânia Franco, Jéssica Geronutti, Luís Brizola, Letícia Ishimoto, Yasmin Barros, Adriele Costa, Carolina Breda, Caroline Marin, Fernanda Suetake, Paula Azevedo, Sergio de Paiva, Suzana Tanni, Robson Prudente

**Affiliations:** 1 São Paulo State University (Unesp), Medical School, Distrito de Rubião Junior s/n, Botucatu 18618-970, São Paulo, Brazil; maerciosantos13@gmail.com (M.S.); marianadorna@gmail.com (M.D.); lfpb@uninove.edu.br (L.B.); leticia.yumi@uol.com.br (L.I.); yasmin.oliveirabarros@gmail.com (Y.B.); adriele.fog@gmail.com (A.C.); carolinacbreda@hotmail.com (C.B.); carolinesantosmarin@hotmail.com (C.M.); fers4ori@gmail.com (F.S.); schmidt.azevedo@unesp.br (P.A.); sergio.paiva@unesp.br (S.d.P.); suzanapneumo@gmail.com (S.T.); 2 Clinical Hospital of Botucatu Medical School, São Paulo State University (Unesp), Distrito de Rubião Junior s/n, Botucatu 18618-970, São Paulo, Brazil; estefania.franco@unesp.br (E.F.); jessica.gernutt@unesp.br (J.G.)

**Keywords:** dyspnea, fatigue, muscle strength, post-acute COVID-19 syndrome, quality of life

## Abstract

**Background**/**Objectives**: Post-COVID-19 condition can manifest through various symptoms such as dyspnea, cognitive disturbances, and fatigue, with mechanisms related to these symptoms, particularly those related to fatigue, still requiring further clarification. Therefore, our aim was to assess the clinical and physiological variables in patients with post-COVID-19 condition and persistent fatigue. **Methods**: After one year post-COVID-19 infection, the patients underwent a comprehensive evaluation, including a complete blood count, a metabolic panel, complete spirometry, and assessments of dyspnea, quality of life, anxiety and depression, physical capacity, body composition, muscle strength, comorbidities, and medications. The participants were categorized into two groups: G1—fatigue and G2—non-fatigue. **Results**: Seventy-seven patients (53% female; 55 ± 11.8 years) were included, 37 in G1 and 40 in G2. As for clinical markers and symptoms of illness, in those with persistent fatigue symptoms, a greater sensation of dyspnea [BDI score: 7.5 (6–9) vs. 12 (9–12), *p* < 0.001; mMRC score: 1 (1–2) vs. 0 (0–1), *p* = 0.002], worse quality of life [SGRQ total score: 1404 (1007–1897) vs. 497 (274–985); *p* < 0.001], higher levels of anxiety [HADS-A score: 8 (5–9) vs. 3 (0.5–4); *p* < 0.001], and a reduction in peripheral and inspiratory muscle strength [handgrip strength: 34 (28–40) vs. 40 (30–46.5) kgf, *p* = 0.044; MIP: −81 ± 31 vs. −111 ± 33 mmHg, *p* < 0.001)] were observed. **Conclusions**: Those with persistent fatigue exhibited a greater sensation of dyspnea, higher levels of anxiety, reduced peripheral and inspiratory muscle strength, and a greater impairment of quality of life. The severity of fatigue was influenced by the worsening quality of life, heightened anxiety levels, and decreased peripheral muscle strength. Additionally, the worse quality of life was associated with a higher sensation of dyspnea, lower muscle strength, and reduced physical capacity.

## 1. Introduction

On 5 May 2023, the director-general of the World Health Organization (WHO) declared the end of COVID-19 as a Public Health Emergency of International Concern. However, the disease still poses a global threat to health that demands sustainable and comprehensive management, both in its acute phase and in the lingering sequelae it can leave in those affected [1].

Currently, the severity and mortality associated with COVID-19 have been altered due to advances in disease treatment, including a well-guided vaccination regimen. However, even after the acute phase of COVID-19, some individuals continue to experience symptoms such as dyspnea, headache, cough, chest pain, abdominal pain, muscle pain, fatigue, sleep disturbances, cognitive dysfunction, brain fog, anxiety, and diarrhea [2,3,4]. This condition is referred to as post-COVID-19 condition, commonly known as long COVID, as it involves a comprehensive array of physical and neuropsychiatric symptoms that persist for months or even years without any alternative explanation [5,6,7,8].

The causes of post-COVID-19 condition are still not fully understood; however, there is a hypothesis suggesting a relationship between the severity of the disease during the acute phase and the persistence of symptoms in the chronic phase. One theory in this regard suggests that organic damage occurring during the acute phase, as well as the extent of these injuries and the time required for recovery of each system, may play a significant role in the development of the disease [9,10]. Additionally, it is suggested that factors such as age, high body mass index (BMI), female sex, previous hospitalization, and smoking may be some of the risk factors for its development [6,11].

Regarding fatigue, specifically, although it does not have a clear and universally accepted definition, it is agreed that it corresponds to a state caused by effort resulting in changes in strategy or resource use by the affected individual so that less energy is used in a particular task. Furthermore, fatigue can be categorized into various subtypes such as mental, cognitive, psychological, and physical fatigue. In this sense, although some aspects related to mental fatigue have been addressed in this study, we aim to better understand the concepts related to physical fatigue resulting from post-COVID-19 condition [12,13,14].

The mechanisms underlying fatigue in post-COVID-19 condition also require further clarification. Some authors suggest that, on the one hand, its occurrence is related to autonomic dysfunction involving the limbic system and vagus nerve, which would trigger a cascade of inflammatory events resulting in symptoms of fatigue, myalgia, and sleep disturbances [15,16,17,18]. On the other hand, the involvement of cytokines such as IL-1β, IL-6, IL-10, and TNF-α can also disrupt metabolic homeostasis in the musculature leading to muscle loss (specifically, IL-6) as well as alter levels of corticotropin-releasing hormone, adrenocorticotropic hormone, and cortisol in the hypothalamic–pituitary–adrenal axis, resulting in chronic fatigue symptoms and a fibromyalgia-like condition [19,20,21]. Furthermore, angiotensin-converting enzyme 2 has been found to be expressed in skeletal muscles similarly to cardiac muscles, leading to speculation that COVID-19 may directly attack skeletal myocytes and lead to fibromyalgia symptoms. Chronic inflammation at the neuromuscular junction involves mitochondria, leading to sarcolemma damage, muscle fiber atrophy, and muscle fiber damage, also resulting in the development of myopathy and consequent fatigue [22,23].

In this context, this study proposes an innovative approach to understanding fatigue in post-COVID-19 condition, expanding the focus beyond lung function to include variables such as muscle weakness, anxiety, and quality of life. Consequently, due to the various factors that may be involved in the fatigue symptoms, a greater understanding of clinical and physiological variables in these individuals makes it possible to develop more effective strategies for the diagnosis, treatment, and management of post-COVID-19 condition, thus improving the quality of life and prognosis of affected patients. Therefore, our aim was to evaluate clinical and physiological variables in patients with post-COVID-19 condition and persistent fatigue, hypothesizing that patients with fatigue exhibit a higher prevalence of symptoms, comorbidities, and physiological changes. Furthermore, the severity of fatigue may correlate with the extent of these changes, with distinct subgroups possibly displaying specific clinical and physiological profiles.

## 2. Materials and Methods

This was a prospective cross-sectional study that included patients from previous studies [Evaluation of clinical symptoms, respiratory, radiological and metabolomic function in patients who have been hospitalized for coronavirus infection (COVID-19) in the period of a year: multicentric study (Fenix)—CAAE: 31258820.5.1001.5411; approval number: 4.167.646, dated 13 August 2020, and Phase 2/3, randomized, closed, single-blind, three-arm study to evaluate the effect of creatine supplementation on long-term Covid-related fatigue (Fatigue study)—CAAE: 65312822.8.0000.5411; approval number: 5.902.259, dated 14 December 2023] with confirmed diagnosis of COVID-19 treated and monitored at the Clinical Hospital of Botucatu Medical School (HCFMB), in the city of Botucatu, SP, Brazil, from 2020 to 2024.

After one year post-COVID-19 infection, the patients underwent a comprehensive evaluation including a complete blood count, a metabolic panel, complete spirometry, assessment of dyspnea (baseline dyspnea index (BDI) and modified Medical Research Council dyspnea scale (mMRC)), measurement of quality of life (St. George’s Respiratory Questionnaire (SGRQ)), assessment of anxiety and depression (Hospital Anxiety and Depression Scale (HADS)), evaluation of physical capacity (six-minute walk test (6MWT)) [24], measurement of body composition (bioelectrical impedance analysis), measurement of muscle strength (handgrip strength and maximal inspiratory and expiratory pressures (MIP and MEP)) [25], documentation of comorbidities, and review of medications. The participants were categorized into two groups: G1—fatigue and G2—non-fatigue. The fatigue categorization was conducted using the Revised Piper Fatigue Scale (PFS-R), with a cutoff point of ≥4 [26,27].

The mMRC scale assesses dyspnea using a five-point scale based on activities that provoke breathlessness, where higher scores indicate greater dyspnea severity. The BDI evaluates three key aspects: the intensity of tasks causing dyspnea, the effort required to induce dyspnea, and the functional impairment caused by dyspnea. Each item is rated from zero to four, and the total score is the sum of these three items, with lower scores indicating more severe dyspnea [28,29].

The SGRQ comprises three domains: symptoms, which assesses the discomfort caused by respiratory symptoms; impact, which evaluates the overall effect on daily activities and patient well-being; and activities, which measures changes in physical activity. The total score is the sum of these domains, ranging from 0 to 100 points, where 0 indicates no dysfunction and 100 indicates maximum dysfunction. Results are expressed as percentages, with changes exceeding 4% indicating clinically significant differences in each domain [30].

The HADS includes 14 items, with 7 each assessing anxiety (HADS-A) and depression (HADS-D). Each item is scored individually from 0 to 3, resulting in a maximum score of 21 points for each scale. The cutoff scores follow Zigmond and Snaith’s criteria: HADS-A scores of 0 to 8 indicate no anxiety, while scores of 9 or higher indicate the presence of anxiety; HADS-D scores of 0 to 8 indicate no depression, while scores of 9 or higher indicate the presence of depression [31].

Initially developed to assess fatigue in cancer patients, the PFS-R has since been validated for other languages and clinical settings [26,32,33,34,35]. It comprises a multidimensional self-report instrument with 5 open-ended questions and 22 closed-ended items, organized into three dimensions: the behavioral dimension, which assesses functional capacity (personal issues, social activities, and sexual relationships); the affective dimension, aimed at understanding the meaning attributed to fatigue; and the sensory/psychological dimension, exploring components of self-perception, emotional, and cognitive aspects in the presence of fatigue. Each dimension, as well as the total score, is calculated as the average score of its respective items, ranging from 0 to 10. Fatigue presence was considered when the score was equal to or greater than four [36].

The inclusion criteria were patients with a confirmed diagnosis of COVID-19, aged 18 years and older, and with clinical stability at the time of evaluation. The exclusion criteria included preexisting, severe, and uncontrolled organ failure, as well as psychological disorders that could prevent understanding the proposed procedures. Additionally, those who presented complications unrelated to fatigue, did not undergo many of the evaluative instruments, and persisted with tomographic alterations justifying the symptoms presented were excluded.

Descriptive statistics were applied to delineate the characteristics of all participants. Variables with a normal distribution were presented as mean values, standard deviations, medians, and 25–75% percentiles for non-parametric variables. Student’s *t*-test compared normally distributed variables, while the Mann–Whitney test assessed non-normally distributed ones. The chi-squared test scrutinized binary qualitative variables with frequencies greater than 5, while the McNemar test compared proportions within the same group. Relevant correlations were explored using Pearson’s and Spearman’s correlation coefficients. A multiple logistic regression analysis was conducted to assess attributes associated with fatigue, with a model adjusted for the occurrence of fatigue as the dependent variable and covariates including SGRQ total, HADS-A, handgrip strength, mMRC, sex, and smoking history (variables showing significantly different values between groups with and without fatigue). A 5% significance level was applied to all tests. The statistical analyses were performed using Jamovi version 2.3 (The Jamovi project, Sydney, Australia).

## 3. Results

**Figure 1:**
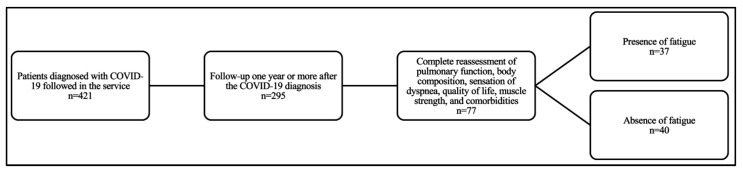
Flow diagram.

In total, 77 patients vaccinated against COVID-19 at the time of evaluation (53% female; 55 ± 11.8 years) were included, 37 in G1 and 40 in G2 (Figure 1). The median time between symptom onset and the assessment of the presence or absence of fatigue was 427 (range: 357–670) days (Table 1).

As for clinical markers and symptoms of illness, a greater sensation of dyspnea, worse quality of life, higher levels of anxiety, and reductions in peripheral and inspiratory muscle strength were observed in those with persistent fatigue symptoms (Table 2).

When these variables were correlated, moderate and/or strong correlations were observed between mMRC and MIP (r = 0.50; *p* < 0.001), grip strength (r = −0.53; *p* < 0.001), and total SGRQ (r = 0.73; *p* < 0.001). Total SGRQ correlated strongly with BDI (r = −0.75; *p* < 0.001), grip strength (r = −0.53; *p* < 0.001), and MIP (r = 0.53; *p* < 0.001). Additionally, although weak, correlations were observed between 6MWD and mMRC (r = −0.48; *p* < 0.001) and total SGRQ (r = −0.40; *p* < 0.001). In the multiple regression analysis, total SGRQ, HADS-A, and handgrip strength may explain the occurrence of fatigue (Table 3).

Regarding comorbidities, it was noted that G1 presented a higher proportion of autoimmune diseases (*p* = 0.010), while G2 had a higher number of cases of systemic arterial hypertension (*p* = 0.017) and diabetes mellitus (*p* = 0.016) (Table 4). When including comorbidities in the logistic regression model, statistically significant results were not observed.

In terms of lung function, only reductions in the values of forced expiratory volume in one second (FEV_1_ (L)) were observed in those with persistent fatigue (Table 5).

Additionally, regarding laboratory markers and previously used medications, except for the platelet count, which was higher in G1 (251.5 ± 50 vs. 216.8 ± 57.2; *p* < 0.007), no significant differences were found between the groups.

## 4. Discussion

We observed that our sample had a higher proportion of women with persistent fatigue symptoms, and those without persistent symptoms were proportionally more hospitalized and required invasive mechanical ventilation (IMV). Unfortunately, we encountered the bias that all patients without fatigue were hospitalized, which, consequently, increased the proportion of those who required IMV, making these two parameters (hospitalization and IMV use) difficult to analyze impartially in our sample. However, the higher proportion among females aligns with some studies related to the topic [12,37,38].

Some authors suggest that women, indeed, report higher levels of fatigue than men. These findings may be attributed to both biological factors, such as changes during the menstrual cycle and pregnancy, and social factors, such as the predominant role of women as primary caregivers for children and household responsibilities, which are still common in various societies [12,37,38]. More specifically, regarding fatigue related to post-COVID-19 condition, a recent cohort study by Saito et al. [2] corroborated our findings, also noting that this condition disproportionately affected more women than men (approximately 70% of the sample).

Together with fatigue, the sensation of dyspnea is one of the most reported symptoms among patients with post-COVID-19 condition, and it can persist for years [3,11,39,40]. A meta-analysis by Ma et al. [40] involving 10,945 cases of COVID-19 demonstrated that, although mild, the sensation of dyspnea was one of the most reported symptoms, like Njøten et al. [39], who, although in a smaller sample (n = 65), found that 66% of the patients reported a sensation of dyspnea, even though 87% of the individuals had normal lung function. A similar finding was observed in our study, as even with the presence of the sensation of dyspnea, lung function, except for FEV_1_ in liters, did not differ between the groups.

Other studies also did not find significant changes in lung function after the acute phase of COVID-19, and when observed, the changes were more related to the diffusing capacity of the lungs for carbon monoxide (DLCO) than to changes related to lung flow and/or volumes [41,42]. In this sense, Alahmari et al. [41] found that after one year of COVID-19 infection, the evaluated subjects had relatively preserved lung function because the observed changes were more related to the percentage of predicted DLCO, which was significantly lower compared with the control group. A similar finding was observed by Sanhueza et al. [42] who, in a sample of 60 individuals, found that 23% had long-term pulmonary dysfunction, characterized by DLCO alterations and chest tomography. 

Still, regarding lung function, it is worth noting that although the FEV_1_(L) was lower in those with persistent fatigue, its values were above 80% of the reference value and above the lower limit predicted in both groups, suggesting, in our sample, that the sensation of fatigue may have been due to causes other than pulmonary ones.

From this perspective, we also observed that patients with persistent fatigue showed lower inspiratory and peripheral muscle strength. Regarding this, it is known that COVID-19 negatively impacts various organs and systems, including the skeletal muscle tissue, resulting in reduced mobility, weakness, muscle fatigue, and overall physical performance impairment. This is because, in addition to the direct injury mediated by SARS-CoV-2, the musculoskeletal tissue can also be affected by other factors, such as systemic inflammatory processes, electrolyte imbalances, severe myopathy, use of specific medications (such as corticosteroids), and hypoxia [43,44].

Similar to our findings, a study by Ramírez-Vélez et al. [45] observed a significant reduction in peripheral muscle strength among 99 patients with post-COVID-19 condition compared with controls, with this reduction being primarily associated with the appendicular lean mass index. In our sample, although we observed a tendency toward lower lean mass in those with fatigue, the comparison between groups did not show a significant difference. Similarly, Mittal et al. [46] observed a reduction in handgrip strength in patients with type 2 diabetes mellitus and post-COVID-19 condition. 

Like peripheral muscles, it is suggested that respiratory muscle dysfunction in patients with post-COVID-19 condition is a central aspect of COVID-19 sequelae [47,48]. Hennigs et al. [47] found that in a sample of 67 individuals, 88% were below age-specific reference values, predominantly in those who required hospitalization and in female patients. In our sample, although we observed a significant reduction in inspiratory muscle strength, the predicted values were above 80% in both groups.

Regarding physical capacity, previous studies have found greater reductions following COVID-19 infection [48,49,50], and in our sample, even though there was a tendency for lower values, patients with persistent fatigue symptoms did not show statistically significant differences in the 6 min walk test (6MWT). When analyzing the percentage of predicted values for the population, both groups had median values above 80% of the reference values. However, in absolute terms, we observed that patients with persistent fatigue walked an average of 37 m less, which may represent a clinically significant difference in these individuals. 

Specific data regarding 6MWT variations in post-COVID-19 condition are still scarce. However, in other chronic lung diseases such as chronic obstructive pulmonary disease (COPD), recent studies have shown shorter distances as cutoff points for worse outcomes in this population, with values ranging between 35 m, 30 m, and even 25 m [51,52,53]. The mechanisms related to the reduction in physical capacity still require further investigation. In this regard, Wong et al. [50], in a cohort including 225 COVID-19 patients, found an association between a shorter distance walked in the 6MWT, exertional dyspnea, and hypoxemia. Although we did not analyze exertional dyspnea in our sample, when correlating the distance walked in the 6MWT with the sensation of dyspnea at rest, even weakly, we observed an inversely proportional correlation between 6MWT and mMRC, suggesting a greater sensation of dyspnea in those who walked less.

In addition, we found that patients with persistent fatigue exhibited higher levels of anxiety, significantly influencing the occurrence of fatigue. Consistent with previous studies, we reinforce the hypothesis that individuals infected with SARS-CoV-2 are at a higher risk of anxiety and depression [54,55,56,57]. The exact mechanisms underlying the development of these conditions also need further exploration. However, some evidence suggests multifactorial aspects related to systemic inflammation, cytokine release, neuroinflammation, and microvascular thrombosis resulting from COVID-19 [54,55].

Besides that, we did not observe significant differences in either the medications used or in most of the laboratory parameters analyzed, except for the platelet count, which was significantly higher in those with persistent fatigue symptoms. However, despite this, although thrombocytosis has been discussed in COVID-19 patients, in our sample, the values found were within the normal range in both groups [58].

Regarding the comorbidities presented, we do not believe they may have influenced our findings, as patients with persistent fatigue only showed a higher proportion of autoimmune diseases. However, it is important to note that these patients represented only 16% of the total sample of those with persistent fatigue symptoms.

As expected, these findings were reflected in the quality of life of these subjects. Patients with persistent fatigue exhibited greater impairment in quality of life, which correlated with a greater sensation of dyspnea, lower respiratory and peripheral muscle strength, and reduced physical capacity. Previous studies have shown that COVID-19 can continue to affect the quality of life of those affected months or even years after the acute phase, which may be more pronounced in those with post-COVID-19 condition and correlate with other symptoms, clinical markers of disease, or even specific characteristics of each individual such as sex and age [57,59]. In this sense, Huang et al. [57] found that after two years from the acute phase, in a sample of 1192 patients with post-COVID-19 condition who required hospitalization, in addition to a lower quality of life, they presented a worse exercise capacity, more mental health abnormalities, and increased use of health services compared with those without post-COVID-19 sequelae. Furthermore, AlRasheed et al. [16] observed in a study involving 400 patients with prior COVID-19 that factors such as male gender, older age, higher BMI, the presence of diabetes mellitus, higher educational levels, and smoking were associated with a lower quality of life. It is important to note that these factors may not only affect an individual’s well-being but could also impact their ability to participate in productive work-related activities.

The underlying mechanisms of persistent fatigue after COVID-19 remain unclear; however, we observed indications that extrapulmonary physiological sequelae play a role in this process. This is evidenced by changes in clinical markers and the persistence of symptoms, even when lung function is within normal limits. However, it is important to emphasize that at this moment, we are only suggesting the possible contribution of these factors, without establishing a definitive cause-and-effect relationship, and the data obtained should not be generalized.

The variability in symptoms is reflected in the treatment of the disease. Currently, the proposed treatment involves the use of various medications, alternative medicine approaches, cognitive-behavioral therapy, pulmonary rehabilitation, and physical exercises; however, high-quality evidence-based studies are lacking regarding the effectiveness of treatments and interventions for fatigue in patients with post-COVID-19 condition, highlighting the difficult recovery process and encouraging the search for new interventions, including to reduce the disease’s impact time [10,60].

Finally, we highlight some limitations of the present study that require clarification. Firstly, the subjective assessment of fatigue using a scale can reflect each participant’s individual perception, which may vary based on various factors such as emotional state, previous experiences, and cultural context. Secondly, the sample size was not robust, which may compromise the generalization of the results. Additionally, the control sample mainly consisted of individuals who required hospitalization during the acute phase of the infection, which reflects the severity of the disease in this group. Lastly, correlations between variables, such as muscle weakness and possible underlying mechanisms, such as inflammation and hypoxia, were also not explored, highlighting the need for additional studies for a more comprehensive understanding of the long-term effects of COVID-19.

## 5. Conclusions

When assessing clinical and physiological variables in patients with post-COVID-19 condition and persistent fatigue symptoms, those with persistent fatigue exhibited a greater sensation of dyspnea, higher levels of anxiety, reduced peripheral and inspiratory muscle strength, and a greater impairment of quality of life. Furthermore, the severity of fatigue was influenced by the worsening quality of life, heightened anxiety levels, and decreased peripheral muscle strength. Additionally, the worse quality of life in these individuals was associated with a higher sensation of dyspnea, lower muscle strength, and reduced physical capacity, without significant alterations in laboratory parameters and pulmonary function.

## Figures and Tables

**Table 1:** General characterization of the sample.

Variable	Fatigue (n = 37)	Non-Fatigue (n = 40)	*p*-Value
Sex, f/m	25/12	16/24	0.015 ^a^
Age, years	53.7 ± 11.7	56.2 ± 11.8	0.351 ^b^
BMI, kg/m^2^	30.5 (27.3–34.9)	32.2 (28.9–35.4)	0.429 ^c^
Time of symptoms, days ^1^	621 ± 289	406 ± 99.4	<0.00 ^b^
Hospitalization, y/n	17/20	40/0	<0.00 ^a^
IMV, y/n	4/33	12/28	0.03 ^a^
Smoking history, y/n	14/23	18/22	0.524 ^c^

Values are expressed as means ± standard deviation, medians (interquartile range 25–75%), or proportions between groups. BMI: body mass index; kg/m^2^: kilograms per square meter; IMV: invasive mechanical ventilation. ^1^ Time between COVID-19 infection and assessment of the presence or absence of fatigue. ^a^ Chi-squared or Fisher’s exact; ^b^ Student’s *t*-test; ^c^ Mann–Whitney U test.

**Table 2:** Symptoms and health status assessment.

Variable	Fatigue (n = 37)	Non-Fatigue (n = 40)	*p*-Value
mMRC, score	1 (1–2)	0 (0–1)	0.002 ^a^
BDI, score	7.5 (6–9)	12 (9–12)	<0.001 ^a^
SGRQ symptom, %	213 (110–316)	173 (42–244)	0.119 ^a^
SGRQ activity, %	720 ± 281	337 ± 318	<0.001 ^b^
SGRQ impact, %	555 (320–788)	65 (0–226)	<0.001 ^a^
SGRQ total, score	1404 (1007–1897)	497 (274–985)	<0.001 ^a^
HADS-A, score	8 (5–9)	3 (0.5–4)	<0.001 ^a^
HADS-D, score	5 (2–10)	3 (0–4)	0.054 ^a^
6MWT, m	440 ± 91	477 ± 87	0.075 ^b^
6MWT, % of predicted	84 (70.5–93)	82 (74–95)	0.567 ^a^
Handgrip strength, kgf	34 (28–40)	40 (30–46.5)	0.044 ^a^
MIP, mmHg	−81 ± 31	−111 ± 33	<0.001 ^b^
MIP, % of predicted	87 ± 32	112 ± 27	<0.001 ^b^
MEP, mmHg	95 (70–118)	110 (87.5–143)	0.074 ^a^
MEP, % of predicted	103 ± 30	110 ± 25	0.249 ^b^
Weight, kg	79.6 (68.7–95.3)	85.9 (75.3–99.1)	0.169 ^a^
Height, m	1.62 ± 0.08	1.65 ± 0.09	0.289 ^b^
LBM, kg	26.8 ± 6.4	29.4 ± 6.2	0.076 ^b^
BFM, kg	36.0 ± 15.6	35.7 ± 11.6	0.925 ^b^

Values are expressed as means ± standard deviation or medians (interquartile range: 25–75%). mMRC: modified Medical Research Council dyspnea scale; BDI: baseline dyspnea index; SGRQ: St. George’s Respiratory Questionnaire; HADS-A: hospital anxiety and depression scale—anxiety; HADS-D: hospital anxiety and depression scale—depression; 6MWT: 6 min walk test; m: meters; kgf: kilogram-force; MIP: maximal inspiratory pressure; mmHg: millimeters of mercury; MEP: maximal expiratory pressure; kg: kilogram; LBM: lean body mass; BFM: body fat mass. ^a^ Mann–Whitney U test or ^b^ Student’s *t*-test.

**Table 3:** Results of the logistic regression analysis using the occurrence of fatigue as a dependent variable.

Variable	Estimate	SE	Z	*p*	Odds	95% Confidence Interval	
						Lower	Upper
Intercept	−7.41837	2.6439	−2.806	0.005	6.00 × 10^−4^	3.37 × 10^−6^	0.107
SGRQ total, score	0.00270	9.52 × 10^−4^	2.840	0.005	1.003	1.0008	1.005
HADS-A, score	0.26889	0.0996	2.699	0.007	1.309	1.0764	1.591
Handgrip strength, kgf	0.133894	0.0667	2.033	0.042	1.145	1.0049	1.305
mMRC, score	−0.33894	0.5794	−0.585	0.559	0.713	0.2289	2.218
Sex Male–female (reference)	−1.71506	1.2099	−1.417	0.156	0.156	0.0168	1.928
Smoking history Yes–no (reference)	−0.63112	0.8772	−0.719	0.472	0.472	0.0953	2.969

Nagelkerke r^2^ = 0.563. SGRQ: St. George’s Respiratory Questionnaire; HADS-A: hospital anxiety and depression scale—anxiety; kgf: kilogram-force; mMRC: modified Medical Research Council dyspnea scale. Estimates present the Log odds of presence of fatigue (absence of fatigue as a reference of model).

**Table 4:** Comorbidities presented by patients with and without fatigue.

Variable	Fatigue (n = 37)	Non-Fatigue (n = 40)	*p*-Value
Asthma, y/n	3/34	3/37	1.000
COPD, y/n	2/35	2/38	1.000
Hypertension, y/n	14/23	26/14	0.017
Other heart diseases, y/n	4/33	3/37	0.705
Diabetes mellitus, y/n	5/32	15/25	0.016
Dyslipidemia, y/n	3/34	2/38	0.580
Obesity, y/n	20/17	26/14	0.328
Osteoporosis, y/n	1/36	0/40	0.481
Autoimmune disease, y/n	6/31	0/40	0.010
Chronic renal disease, y/n	2/35	1/39	0.605
Chronic liver disease, y/n	3/34	0/40	0.106
Neoplasm, y/n	1/36	1/39	1.000
Stroke, y/n	0/37	3/37	0.241

Values are expressed as proportions between groups. Chi-squared or Fisher’s exact test.

**Table 5:** Pulmonary function tests of patients with and without fatigue.

Variable	Fatigue (n = 37)	Non-Fatigue (n = 40)	*p*-Value
FVC, L	3.0 ± 0.7	3.3 ± 0.7	0.057 ^a^
FVC, %	85.6 ± 14.9	88.0 ± 13.4	0.459 ^a^
FEV_1_, L	2.45 ± 0.59	2.79 ± 0.69	0.022 ^a^
FEV_1_, %	85.6 ± 16.9	90.7 ± 14.2	0.160 ^a^
FEV_1_/FVC, L	0.83 (0.80–0.85)	0.84 (0.79–0.85)	0.281 ^b^
TLC, L	4.38 ± 1.06	4.70 ± 1.07	0.234 ^a^
TLC, %	85.0 (73–92.5)	85.0 (74–95.5)	0.600 ^b^
RV, L	1.10 (0.92–1.52)	1.32 (1.11–1.69)	0.113 ^b^
RV, %	65.0 (52.5–81.5)	70.5 (56.5–87.3)	0.569 ^b^
DLCO, mL/mmHg/min	21.8 ± 4.88	23.4 ± 6.78	0.277 ^a^
DLCO, %	82.3 ± 11.7	82.6 ± 16.5	0.927 ^a^
DLCO/VA, mL/mmHg/min/L	5.41 (4.45–5.64)	5.14 (4.65–5.64)	0.775 ^b^
DLCO/VA, %	99.3 ± 15.7	97.1 ± 14.7	0.570 ^a^

Values are expressed as means ± standard deviation or medians (interquartile range: 25–75%). FVC: forced vital capacity; L: liter; FEV_1_: forced expiratory volume in the first second; TLC: total lung capacity; RV: residual volume; DLCO: diffusing capacity of the lungs for carbon monoxide; mL: milliliter; mmHg: millimeters of mercury; DLCO/VA: diffusing capacity divided by the alveolar volume. ^a^ Student’s *t*-test; ^b^ Mann–Whitney U test.

## Data Availability

The datasets used and/or analyzed during the current study are available from the corresponding author upon reasonable request.
